# Immunization with SARS Coronavirus Vaccines Leads to Pulmonary Immunopathology on Challenge with the SARS Virus

**DOI:** 10.1371/journal.pone.0035421

**Published:** 2012-04-20

**Authors:** Chien-Te Tseng, Elena Sbrana, Naoko Iwata-Yoshikawa, Patrick C. Newman, Tania Garron, Robert L. Atmar, Clarence J. Peters, Robert B. Couch

**Affiliations:** 1 Department of Microbiology and Immunology, The University of Texas Medical Branch, Galveston, Texas, United States of America; 2 Center for Biodefense and Emerging Disease, The University of Texas Medical Branch, Galveston, Texas, United States of America; 3 Department of Medicine, Baylor College of Medicine, Houston, Texas, United States of America; 4 Department of Molecular Virology and Microbiology, Baylor College of Medicine, Houston, Texas, United States of America; German Primate Center, Germany

## Abstract

**Background:**

Severe acute respiratory syndrome (SARS) emerged in China in 2002 and spread to other countries before brought under control. Because of a concern for reemergence or a deliberate release of the SARS coronavirus, vaccine development was initiated. Evaluations of an inactivated whole virus vaccine in ferrets and nonhuman primates and a virus-like-particle vaccine in mice induced protection against infection but challenged animals exhibited an immunopathologic-type lung disease.

**Design:**

Four candidate vaccines for humans with or without alum adjuvant were evaluated in a mouse model of SARS, a VLP vaccine, the vaccine given to ferrets and NHP, another whole virus vaccine and an rDNA-produced S protein. Balb/c or C57BL/6 mice were vaccinated IM on day 0 and 28 and sacrificed for serum antibody measurements or challenged with live virus on day 56. On day 58, challenged mice were sacrificed and lungs obtained for virus and histopathology.

**Results:**

All vaccines induced serum neutralizing antibody with increasing dosages and/or alum significantly increasing responses. Significant reductions of SARS-CoV two days after challenge was seen for all vaccines and prior live SARS-CoV. All mice exhibited histopathologic changes in lungs two days after challenge including all animals vaccinated (Balb/C and C57BL/6) or given live virus, influenza vaccine, or PBS suggesting infection occurred in all. Histopathology seen in animals given one of the SARS-CoV vaccines was uniformly a Th2-type immunopathology with prominent eosinophil infiltration, confirmed with special eosinophil stains. The pathologic changes seen in all control groups lacked the eosinophil prominence.

**Conclusions:**

These SARS-CoV vaccines all induced antibody and protection against infection with SARS-CoV. However, challenge of mice given any of the vaccines led to occurrence of Th2-type immunopathology suggesting hypersensitivity to SARS-CoV components was induced. Caution in proceeding to application of a SARS-CoV vaccine in humans is indicated.

## Introduction

Severe acute respiratory syndrome (SARS) emerged in Guangdong, People's Republic of China, in late 2002, and spread to other countries in Asia and to Canada in the ensuing months [1]–[3]. Infection control efforts brought the infection under control by mid-2003 [4]. More than 8000 cases, including almost 800 deaths, were reported during the outbreak period [4]. Increasing age and comorbidity were risk factors for severe disease and death [5], [6], [7]. Since 2003, only sporadic cases have been reported; however, the possibility that SARS outbreaks could reemerge naturally or be deliberately released is a public health concern.

SARS is caused by a Coronavirus (SARS-CoV) [8], [9]. Limited data are available about the ecology of SARS-CoV, but bats are thought to be the animal reservoir for the virus which may be transmitted to small mammals with exposure to these small animals as the source of human infections [10]. The clinical disease is similar to other severe acute respiratory infections, including influenza; the SARS case definition includes clinical, epidemiologic, and laboratory criteria [11], [12]. A number of therapeutic efforts were employed for the disease in Asia and in Canada; however, no treatment of clear value was identified. Animal models were developed using mice, hamsters, ferrets and nonhuman primates, and efforts to identify useful treatments and effective vaccines are ongoing.

Vaccine candidates for preventing SARS have been developed by various groups and include inactivated whole virus, spike (S) protein preparations, virus-like particles (VLPs), plasmid DNA and a number of vectors containing genes for SARS-CoV proteins [13]–[28]. Phase I studies in humans have been conducted with a whole virus vaccine and a DNA vaccine [29]–[30].

An early concern for application of a SARS-CoV vaccine was the experience with other coronavirus infections which induced enhanced disease and immunopathology in animals when challenged with infectious virus [31], a concern reinforced by the report that animals given an alum adjuvanted SARS vaccine and subsequently challenged with SARS-CoV exhibited an immunopathologic lung reaction reminiscent of that described for respiratory syncytial virus (RSV) in infants and in animal models given RSV vaccine and challenged naturally (infants) or artificially (animals) with RSV [32], [33]. We and others described a similar immunopathologic reaction in mice vaccinated with a SARS-CoV vaccine and subsequently challenged with SARS-CoV [18], [20], [21], [28]. It has been proposed that the nucleocapsid protein of SARS-CoV is the antigen to which the immunopathologic reaction is directed [18], [21]. Thus, concern for proceeding to humans with candidate SARS-CoV vaccines emerged from these various observations.

The studies reported here were conducted to evaluate the safety, immunogenicity, and efficacy of different SARS-CoV vaccines in a murine model of SARS.

## Materials and Methods

### Tissue Cultures and Virus

Vero E6 tissue cultures [obtained from The American Type Culture Collection (ATCC), CRL:1586] were grown in Dulbecco's modified minimum essential medium (DMEM) supplemented with penicillin (100 units/ml), streptomycin (100 µg/ml), 0.2% sodium bicarbonate and 10% fetal bovine serum (FBS). The Urbani strain of SARS-CoV was obtained from T.G. Ksiazek at the Centers for Disease Control and Prevention (Atlanta, GA), and a working stock of this virus was prepared by serially passaging a portion of the seed virus three times (p3) in Vero E6 cultures. The culture fluid from infected cells was clarified by low-speed centrifugation, filtered through a 0.45 µm filter, aliquoted, and stored at −80°C.

### Vaccines

Four different SARS-CoV vaccines were evaluated in these studies (Table 1). Two whole virus vaccines were evaluated; one was prepared in Vero tissue cultures, zonal centrifuged for purification, and double-inactivated with formalin and UV irradiation, the DI vaccine (DIV); it was tested with and without alum adjuvant [16]. The other whole virus vaccine was prepared in Vero cells, concentrated, purified, inactivated with beta propiolactone and packaged with alum adjuvant (BPV) [13]. A recombinant DNA spike (S) protein vaccine (SV) was produced in insect cells and purified by column chromatography was tested with and without alum adjuvant [17]. The fourth vaccine (the VLP vaccine) was a virus-like particle vaccine prepared by us as described previously; it contained the SARS-CoV spike protein (S) and the Nucleocapsid (N), envelope (E) and membrane (M) proteins from mouse hepatitis coronavirus (MHV) [20].

**Table 1:** Experimental Groups for Evaluation of SARS Coronavirus Vaccines.

Group	Exp 1^1^Vaccine Comparisons	Exp 2^1^Higher SV Dosage plus DIV and BPV Comparisons	Exp 3^1^^,^ 3Mouse and Vaccine Specificity
1	DIV/1 µg^2^	PBS	PBS-PBS
2	DIV/0.5 µg	Live virus	PBS
3	DIV/0.25 µg	SV/9 µg	Live virus
4	DIV/0.125 µg	SV/3 µg	Flu vaccine
5	DIV/1 µg + alum	SV/1 µg	DIV/1 µg
6	DIV/0.5 µg + alum	SV/9 µg + alum	DIV/1 µg + alum
7	DIV/0.25 µg + alum	SV/3 µg + alum	BPV/undil + alum
8	DIV/0.125 µg + alum	SV/1 µg + alum	PBS-PBS
9	SV/2 µg^2^	DIV/1 µg	PBS
10	SV/1 µg	DIV/0.25 µg (50 µl)	Live virus
11	SV/0.5 µg	DIV/1 µg + alum	Flu vaccine
12	SV/0.25 µg	DIV/0.25 µg + alum (50 µl)	DIV/1 µg
13	SV/2 µg + alum	BPV/undil + alum^2^	DIV/1 µg + alum
14	SV/1 µg + alum	BPV/undil + alum (25 µl)	BPV/undil + alum
15	SV/0.5 µg + alum		
16	SV/0.25 µg + alum		
17	VLP/2 µg^2^		
18	VLP/2 µg + alum		
19	Alum		
20	PBS		

1 Design = All experiments in Balb/c mice except as noted in Exp 3. Each group contained 12–13 mice; all were given 100 µl of vaccine IM at dosages with or without alum as indicated on days 0 and 28 except as noted. Five mice in each group were sacrificed on day 56 for serum antibody; remaining mice were given 10^6^ TCID_50_ of SARS-CoV intranasal on day 56 and sacrificed on day 58 for virus and lung histology.

2 DIV/dosage = Vaccine DIV = Zonal centrifuge purified doubly inactivated (formalin and UV) whole virus SV/dosage = Vaccine SV = Recombinant baculovirus expressed S glycoprotein of SARS-CoV VLP/dosage = Vaccine VLP = Virus-like particles containing SARS-CoV S glycoprotein and E, M, and N proteins from mouse hepatitis coronavirus BPV/dosage = Vaccine BPV = Purified beta propiolactone inactivated whole virus plus alum.

3 Experiment 3 = Groups 1 to 7 were Balb/c mice; groups 8 to 14 were C57BL/6 mice. Flu vaccine was licensed trivalent 2009-10 formulation of high dosage vaccine (60 µg of HA of each strain). Groups 1 and 8 were given PBS (placebo) and challenged with PBS; all others were challenged with live SARS-CoV.

### Animals

Six- to eight-week-old, female Balb/c and C57BL/6 mice (Charles River Laboratory, Wilmington, MA), were housed in cages covered with barrier filters in an approved biosafety level 3 animal facility maintained by the University of Texas Medical Branch (UTMB) at Galveston, Texas. All of the experiments were performed using experimental protocols approved by the Office of Research Project Protections, Institutional Animal Care and Use Committee (IACUC), University of Texas Medical Branch and followed National Institutes of Health and United States Department of Agriculture guidelines.

### Study Design

Three different experiments, performed for comparing different vaccines, are reported here. Adjuvanted (alum) and non-adjuvanted (PBS) vaccines were obtained from the NIH/BEI resource. Groups of mice (N = 12–13 per group) were administered various dosages of each vaccine intramuscularly (IM) on days 0 and 28; mice given only PBS, alum, trivalent inactivated influenza vaccine or live SARS-CoV were included as controls in various experiments. On day 56, five mice from each group were sacrificed for assessing serum neutralizing antibody titers and lung histopathology; the remaining seven or eight mice in each group were challenged with 10^6^TCID_50_/60 µl of SARS-CoV intranasally (IN). Challenged mice were euthanized on day 58 for determining virus quantity and preparing lung tissue sections for histopathologic examination.

### Neutralizing Antibody Assays

Mice were anesthetized with isoflurane and then bled from the retro-orbital sinus plexus. After heat inactivation at 56°C for 30 minutes, sera were stored at −80°C until tested. Assays for virus-specific neutralizing antibodies were performed on serial 2-fold diluted samples of each serum using 2% FBS-DMEM as the diluent in 96-well tissue culture plates (Falcon 3072); the final volume of the serially diluted samples in each well was 60 µl after addition of 120 TCID_50_ of SARS-CoV in 60 µl into each well. The beginning dilution of serum was 1∶20. The dilutions were incubated for 45–60 minutes at room temperature; then 100 µl of each mixture was transferred into duplicate wells of confluent Vero E6 cells in 96-well microtiter plates. After 72 hours of incubation, when the virus control wells exhibited advanced virus-induced CPE, the neutralizing capacity of individual serum samples were assessed by determining the presence or absence of cytopathic effect (CPE). Neutralizing antibody titers were expressed as the reciprocal of the last dilution of serum that completely inhibited virus-induced CPE.

### Collection and Processing of Lungs for Histology and Virus Quantity

Two days post SARS-CoV challenge, mice were euthanized and their lungs were removed. Lung lobes were placed in 10% neutral buffered formalin for histological examination and immunohistochemistry (IHC), as described previously [34], [35]. For virus quantitation, the remaining tissue specimen was weighed and frozen to −80°C. Thawed lung was homogenized in PBS/10% FBS solution using the TissueLyser (Qiagen; Retsch, Haan, Germany). The homogenates were centrifuged and SARS-CoV titers in the clarified fluids were determined by serial dilution in quadruplicate wells of Vero E6 cells in 96-well plates. Titers of virus in lung homogenates were expressed as TCID_50_/g of lung (log_10_); the minimal detectable level of virus was 1.6 to 2.6 log_10_ TCID_50_ as determined by lung size.

### Histopathology

Evaluations for histopathology were done by pathologists masked as to the vaccine/dosage of each specimen source; numeric scores were assigned to assess the extent of pathologic damage and the eosinophilic component of the inflammatory infiltrates.

### Statistical Analysis

Neutralizing antibody titers, lung virus titers, histopathologic lesion score and eosinophilic infiltration scores were averaged for each group of mice. Comparisons were conducted using parametric and nonparametric statistics as indicated.

## Results

### Experiments

The three experiments performed, vaccines and dosages used and controls for each experiment are shown in Table 1. The vaccines were evaluated for immunogenicity and efficacy; however, because of the previous report of immunopathology on challenge of ferrets and nonhuman primates that had been vaccinated with a whole virus adjuvanted vaccine and mice that had been vaccinated with a VLP vaccine, the primary orientation was to assess for immunopathology among animals in relation to type of vaccine, dosage, serum antibody responses, and virus infection. The vaccine preparations were made for human trials so identifying a preparation that was likely to be both safe and protective in humans was desired. The rationale for each experiment is described.

#### Comparison of Vaccines (Experiment 1)

To differentiate between vaccines, three vaccine preparations were simultaneously evaluated, the double-inactivated (formalin and UV) whole virus vaccine (DIV), the rDNA-expressed S protein vaccine (SV), and the previously evaluated chimeric viral-like particle vaccine (VLP) that had led to immunopathology with virus challenge [16], [17], [20].

**Figure 1:**
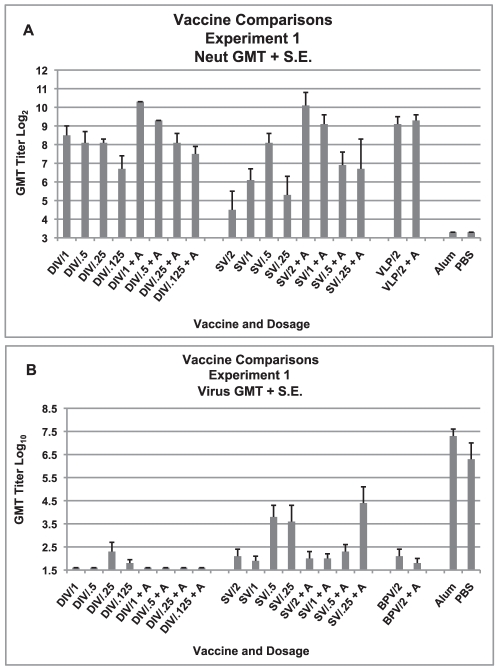
Vaccine Comparisons of Three SARS-CoV Vaccines, Experiment 1. Serum neutralizing (neut) antibody and lung virus titers for each vaccine dosage group. A. Geometric mean serum antibody titer as log_2_ and standard error of the mean (S.E.) on day 56 for each vaccine dosage group. Seven to eight mice per group. Vaccines: double inactivated whole virus (DIV), recombinant S protein (SV), viral-like particle vaccine (VLP), with alum (+A). Five mice per group were given 0.1 ml of vaccine intramuscularly on days 0 and 28. B. Geometric mean virus titer (log_10_ TCID_50_/g) and standard error of the mean (S.E.) in lungs on day 58 (two days after SARS-CoV challenge) for each vaccine dosage group. Analyses: A. GMT with compared to without alum: DIV p>.05, VLP p>.05, SV p = .001. GMT for different vaccine dosage: DIV with alum p = .007, DIV without alum p>.05, SV with alum p = .028, SV without alum p = .01. Multiple regression: GMT increased for alum p = .012 and dosage p<.001, for SV alum only p = .001. B. GMT for all DIV groups not different p>.05, GMT for SV group without alum p .008 and with alum p .023. GMT for VLP group is not different p>.05.

Geometric mean serum neutralizing antibody titers for each group on day 56 are shown in figure 1A. Geometric mean titers for those given a nonadjuvanted or alum adjuvanted vaccine were not different for the double-inactivated whole virus vaccine (DIV), and the VLP vaccine, (p>0.05, student's t-test), but were different for the S protein vaccine (SV) (p = 0.001, student's t test). Geometric mean titers for the different dosage groups given the DI vaccine (DIV) with alum and those for the groups given the S protein vaccine (SV) with or without alum were significantly different (p = 0.007, p = 0.028, and p = 0.01, respectively, Kruskall-Wallis) while the geometric means for those dosage groups given the DI vaccine (DIV) without alum were not (p>0.05, Kruskall-Wallis). In a multiple regression analysis, postvaccination titers for the DI vaccine (DIV) were significantly increased by both alum and higher dosage (for alum, p = 0.012, for dosage, p <0.001); for the S protein vaccine (SV), only alum increased responses (p = 0.001).

Two days after challenge, lungs were obtained from all animals for virus quantitation and histology. CoV titers are shown in figure 1B. Geometric mean lung titers in the alum and PBS control groups were 10^7.3^ and 10^6.3^ TCID_50_/g, respectively. All vaccine groups exhibited lower titers or no detectable virus on day two after challenge. None of the animals given any of the alum-adjuvanted DI vaccine (DIV) dosages and only an occasional animal in the lower dosages of nonadjuvanted vaccine yielded virus (Kruskall-Wallis and Mann Whitney U tests, p>0.05 for all comparisons). All groups given the S protein vaccine (SV) yielded virus after challenge and the differences between groups were significant (p = 0.002 for all groups, p = 0.023 for alum and p = 0.008 for no adjuvant, Kruskall-Wallis); also, geometric mean titers were higher for the groups given lower vaccine dosages. Geometric mean titers for the VLP vaccine groups were similar (p>0.05).

**Figure 2:**
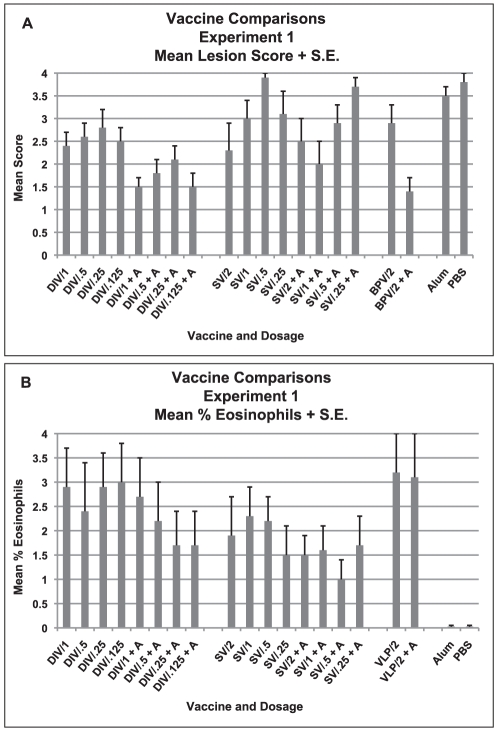
Vaccine Comparisons of Three SARS-CoV Vaccines, Experiment 1. Mean lung cellular infiltration/lesion pathology and percent eosinophils in infiltrates for each vaccine dosage group two days after challenge with SARS-CoV. A. Mean lesion score and standard error of the mean (S.E.) for each vaccine dosage group. All mice exhibited lung histopathology. Scores are mean of scores for seven to eight mice per group. Scoring. 0 – no pathology, 1 and 2 – (1) minimal (2) moderate peribronchiole and perivascular cellular infiltration, 3 and 4 – 1 and/or 2 plus minimal (3) or moderate (4) epithelial cell necrosis of bronchioles with cell debris in the lumen. B. Mean percent eosinophils on histologic evaluation for seven to eight mice in each vaccine dosage group. Mean for each mouse is the mean percent eosinophils on five separate microscopy fields of lung sections. Analyses: A. Mean lesion scores were different p<.001. DIV without alum greater than with alum p = .001, VLP without alum greater than with alum p = .008. Posthoc comparisons: DIV lower than SV p = .001 and controls p<.001 but not VLP p>.05. SV lower than controls p .048. B. Mean percent eosinophils were different p<.001. Mean percent eosinophils lower for DIV with alum than without alum p = .049 and lower for SV with alum than without alum p = .001. Mean percent eosinophils lower for SV than DIV p = .002 or VLP. P = <.001. Mean percent eosinophils greater than controls for DIV, SV and VLP, all three vaccines p<.001.

In the vaccine comparison experiment, lung lesion scores for histopathology were graded for individual animals on a scale of 0 to 4 where 0–2 represented degree of cellular infiltration and 3–4 represented the degree of bronchiolar epithelial cell necrosis and airway cellular debris (figure 2A). As shown, all animals exhibited pathologic changes after challenge including those animals with no measurable virus on day two suggesting virus infection had occurred but was not detectable on day two because of a short duration of infection or neutralization of virus by antibody in the lung during processing. The higher scores (>3) in some groups related primarily to the fact that virus infection had induced inflammatory infiltrates and epithelial cell necrosis with desquamation of the epithelium and collection of cellular debris in airways of these animals. Mean score differences were noted among the various vaccines (p = <0.001, Anova). Those groups given the DI vaccine (DIV) without alum had higher mean scores than did those given DI vaccine (DIV) with alum (p = 0.001, Mann-Whitney U); similarly, the group given the VLP vaccine without alum had a higher mean score than for those given VLP vaccine with alum (p = 0.008, Mann-Whitney U). Post hoc comparisons for the three different vaccines indicated that the DI vaccine (DIV) group overall had lower lesion scores than either the S protein vaccine (SV) group or the alum and PBS control groups (p = 0.001 comparing the DI and S protein vaccines (DIV and SV) and p<0.001 for DIV vs. control groups, Tukey HSD and Dunnett t, respectively), but not the VLP vaccine group (p>0.05, Tukey HSD). The S protein vaccine group (SV) was also lower overall than the control groups (p = 0.048, Dunnett t).

When the characteristics of the infiltrates were compared, animals given alum or PBS exhibited epithelial cell necrosis and peribronchiolar and perivascular mononuclear cell infiltrates consistent with epithelial cell infection and an inflammatory response seen in viral infections. In addition to mononuclear cells, however, infiltrates among vaccinated animals contained neutrophils and eosinophils that were not seen in the lesions of the animals that had been previously given PBS or alum only (figure 2B) suggesting a T helper cell type 2 hypersensitivity reaction; increased eosinophils are a marker for a Th2-type hypersensitivity reaction. Percent eosinophils was lower in these vaccinated animals (mean 1–3.2%) than had been seen in animals given VLP vaccines in the earlier study (mean 13.2±9.6% and 22±9.9% of cells for VLP with PBS or alum, respectively in that study) but no (0%) eosinophils were seen in the lung infiltrates of control animals in this experiment. This pattern of excess eosinophils in cellular infiltrates seen in lung sections from animals given vaccine and not in control animals was as seen in the earlier study with VLP vaccine and those later with other vaccines although the percent eosinophils was lower in this study.

The mean percent eosinophils differed between groups (p<0.001, Anova). Overall, the percent was lower for the groups given the DI and S protein alum adjuvanted vaccines than for the corresponding nonadjuvanted group (p = 0.049 for DIV and 0.001 for SV, Mann-Whitney U). For the vaccines, the eosinophil mean percentages were lower for the S protein vaccine (SV) than for either the DI vaccine (DIV) or VLP vaccine (DIV vs. SV, p = 0.002; VLP vs. SV, p = <0.001, Tukey HSD). Additionally, eosinophil percentages for all three vaccines, including the S protein vaccine, were significantly greater than the controls (SV, DIV and VLP vaccine, p<0.001 for each, Tukey HSD).

#### Higher Dosages of the S Protein Vaccine Plus the bp Inactivated Whole Virus Vaccine, Experiment 2

This experiment was conducted to verify the findings in the initial experiment of a hypersensitivity immunopathologic-like reaction after SARS-CoV challenge of vaccinated animals, to determine if a higher dosage of the S protein vaccine (SV) would suppress infection and still exhibit a similar reaction, and whether the original β propiolactone inactivated whole virus vaccine (BPV) that had shown an immunopathologic-like reaction after challenge of vaccinated ferrets and nonhuman primates exhibited a similar immunopathologic reaction in the mouse model [13], [14]. Additionally, a live virus “vaccination” group was added in this experiment for comparison of challenge results following vaccinations with inactivated vaccines to those following earlier infection.

**Figure 3:**
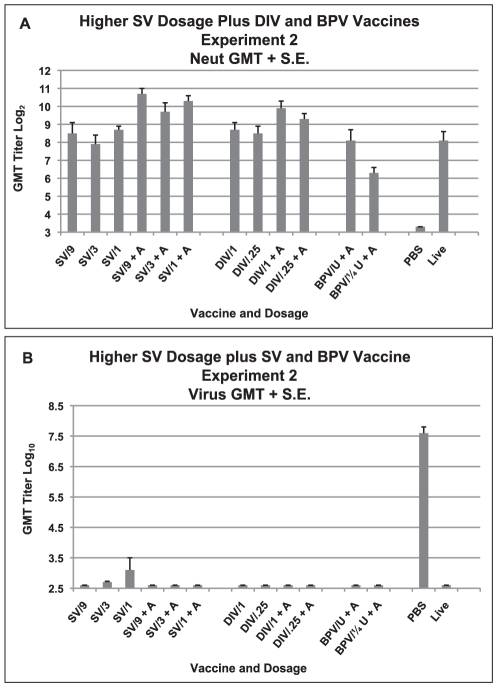
Higher Dosages of SV Vaccine plus DIV and BPV Vaccine Comparisons, Experiment 2. Serum neutralizing (neut) antibody and lung virus titers for each vaccine dosage group. A. Geometric mean serum antibody titer and standard error of the mean (S.E.) on day 56 for each vaccine dosage group. Five mice per group given 0.1 ml of vaccine intramuscularly on days 0 and 28. B. Geometric mean virus titer (log_10_ TCID_50_/g) and standard error of the mean (S.E.) in lungs on day 58 (two days after SARS-CoV challenge) for each vaccine dosage group. Seven to eight mice per group. Vaccines: double inactivated whole virus (DIV), recombinant S protein (SV), β propiolactone inactivated whole virus (BPV) with alum (+A). Analyses: A. GMT with alum greater than without alum: SV p<.001, DIV p = .014. GMT for the two BPV groups are different p = .039. Multiple regression: DIV and SV increased with alum p≤.01, no dosage effect p>.05.

Serum neutralizing antibody responses are shown in figure 3A. The bp inactivated vaccine (BPV), was only available at one dosage with alum so a smaller volume (25 µl) was given to one group for a dosage comparison. Geometric mean titers for the groups given the alum adjuvanted version of the DI and the S protein vaccines were greater than for the unadjuvanted vaccine (DIV P = 0.014, SV p<0.001, student's t test). In multiple regression analysis, titers were also significantly increased after both the DI and S protein vaccines with use of alum (p≤0.01); no dosage effect was noted. The geometric mean neutralizing antibody titers of the two bp inactivated vaccine groups (BPV) were different (p = 0.039, Mann-Whitney U).

Two days after challenge with 10^6^ TCID_50_ of SARS-CoV, titers in mice given PBS varied between 10^7.0^ and 10^8.0^ TCID_50_ per g of tissue; one vaccinated animal in the group given the S protein vaccine (SV) at the 3 µg and the 1 µg dosage without alum yielded virus but all other animals in all other groups were culture negative for virus (figure 3B).

**Figure 4:**
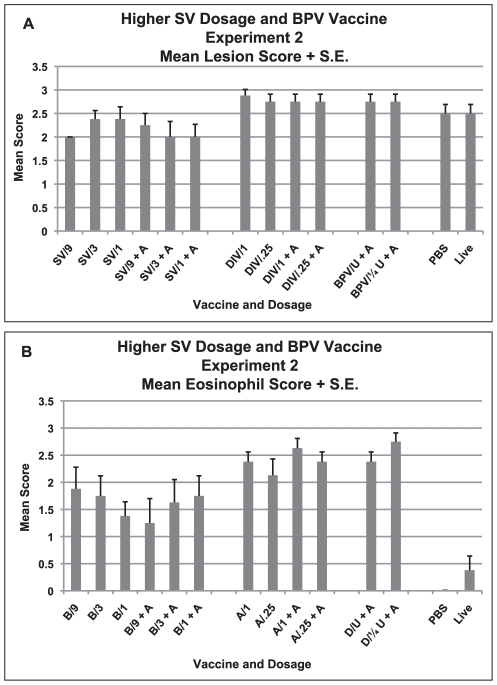
Higher Dosages of SV Vaccine plus DIV and BPV Vaccine Comparisons, Experiment 2. Mean lung cellular infiltration/lesion pathology and mean percent eosinophils in infiltrates for each vaccine dosage group two days after challenge with SARS-CoV. A. Mean lesion score and standard error of the mean (S.E.) for each vaccine dosage group. Scores are mean of scores for seven to eight mice per group. Scoring - 0 - no definite pathology, 1 - mild peribronchiole and perivascular cellular infiltration, 2 - moderate peribronchiole and perivascular cellular infiltration, 3 - severe peribronchiolar and perivascular cellular infiltration with thickening of alveolar walls, alveolar infiltration and bronchiole epithelial cell necrosis and debris in the lumen. Ten to 20 microscopy fields were scored for each mouse lung. B. Mean score and standard error of the mean (S.E.) for eosinophils as percent of infiltrating cells for each vaccine dosage group. Scores are mean of scores for seven to eight mice per group. Scoring: 0 - <5% of cells, 1 - 5–10% of cells, 2 - 10–20% of cells, 3 - >20% of cells. Ten to 20 microscopy fields were scored for each mouse lung. Analyses: A. Mean lesion scores were different p<.001. Mean scores were lower for SV than DIV p<.001 and less than BPV p = .006. B. Mean eosinophil scores were lower for SV than DIV p<.001 and less than BPV p<.001. Eosinophil scores greater for SV than PBS or live virus p<.001.

Shown in figure 4A are the mean lesion scores on histologic evaluations. The scoring system for experiments two and three were developed by a replacement pathologist who preferred a scale of 0 to 3 which corresponded to a judgment of mild, moderate or severe (figure 4A). Mean lesion scores for this grading system overall were significantly different from each other (p<0.001, Anova) and scores were lower for the S protein vaccine than for either of the whole virus vaccines (SV versus DIV and BPV, p<0.001 and p = 0.006, respectively, Tukey HSD). Of interest is that those given live virus and then challenged with live virus two months later exhibited an infiltrative disease severity comparable to the PBS and vaccinated groups despite no detectable virus on day two, again suggesting some degree of infection may have occurred earlier.

The mean eosinophil scores for the lung infiltrations were lower for the S protein vaccine groups [SV vs. DIV p<0.001; SV vs. BPV, p<0.001, Tukey HSD]; however, they were clearly greater than seen in those given PBS or live virus earlier (p<0.001, Tukey HSD) (figure 4B).

**Figure 5:**
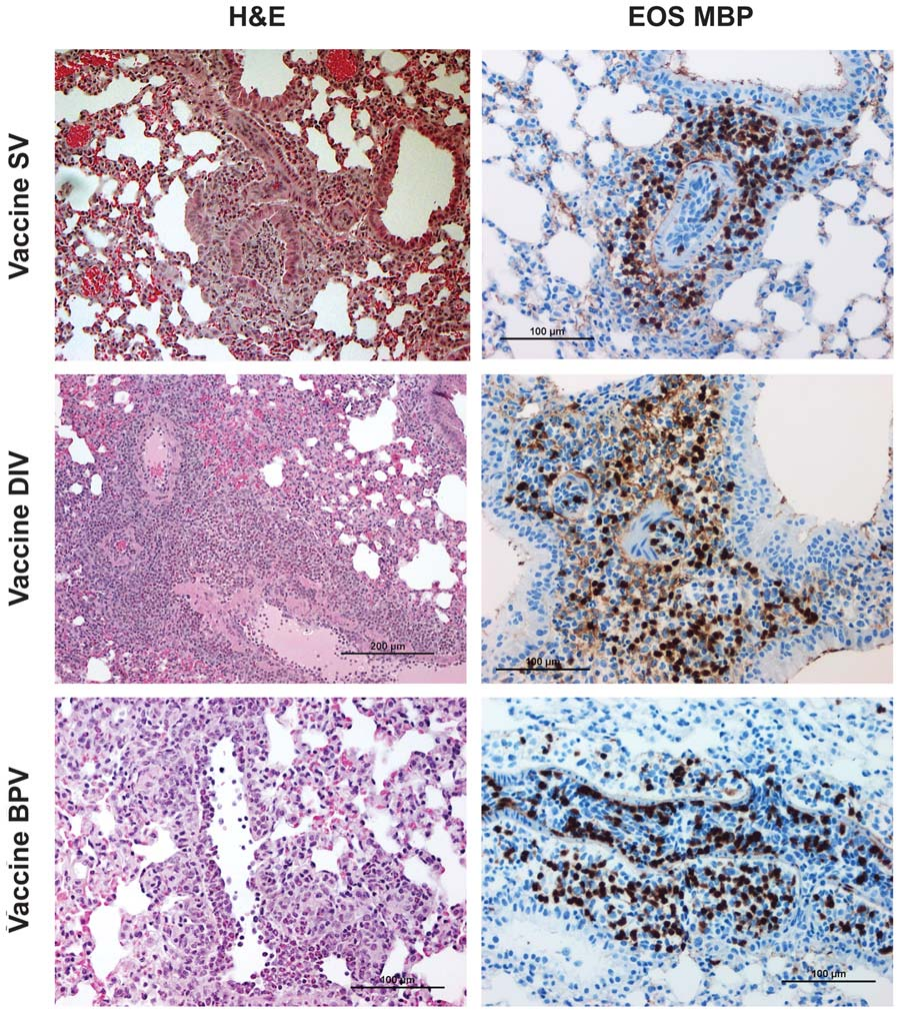
Photographs of Lung Tissue. Representative photomicrographs of lung tissue two days after challenge of Balb/c mice with SARS-CoV that had previously been given a SARS-CoV vaccine. Lung sections were separately stained with hematoxylin and eosin (H&E) and an immunohistochemical protocol using an eosinophil-specific staining procedure with a monoclonal antibody to a major basic protein of eosinophils. DAB chromogen provided the brown eosinophil identity stain. The procedure and antibody were kindly provided by the Lee Laboratory, Mayo Clinic, Arizona. The H&E stain column is on the left and eosinophil-specific major basic protein (EOS MBP) stain column is on the right. Vaccines: double inactivated whole virus (DIV), β propiolactone inactivated whole virus vaccine (BPV). As shown in the images, eosinophils are prominent (brown DAB staining) in all sections examined. Exposure to SARS-CoV is associated with prominent inflammatory infiltrates characterized by a predominant eosinophilic component.

**Figure 6:**
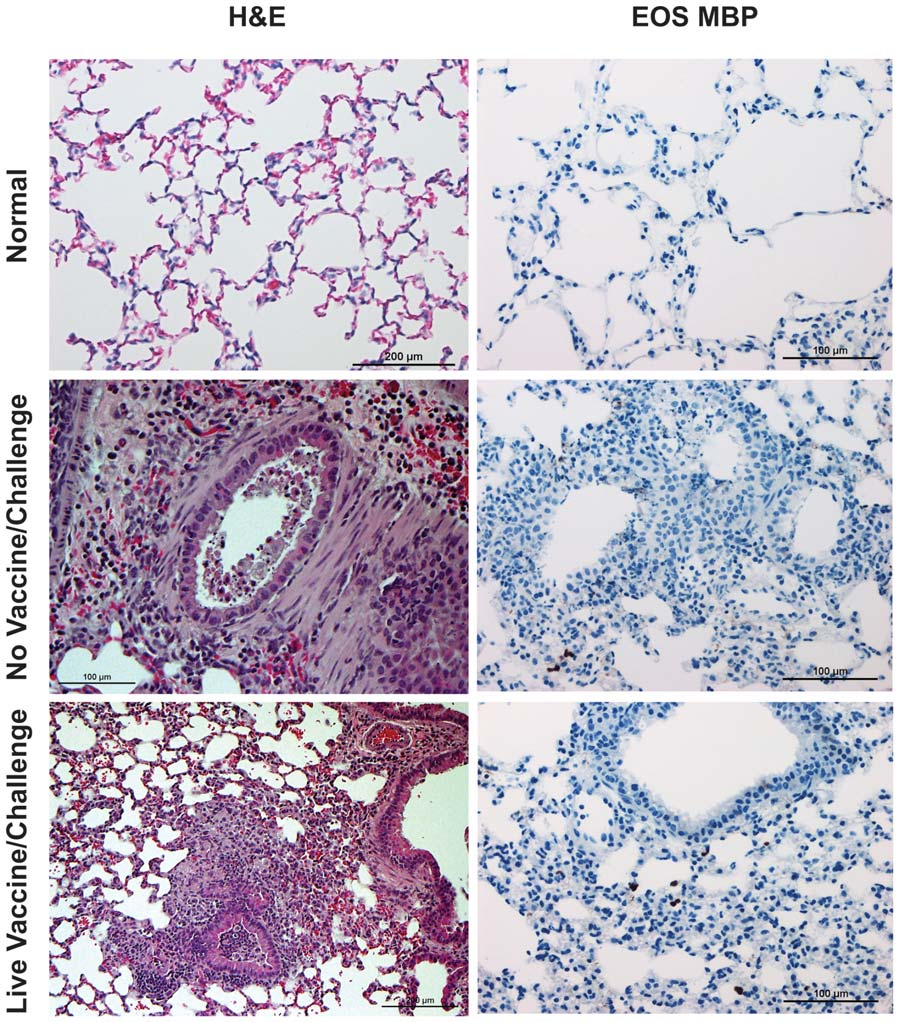
Photomicrographs of Lung Tissue. Representative photomicrographs of lung tissue from unvaccinated unchallenged mice (normal) and from Balb/c mice two days after challenge with SARS-CoV that had previously been given PBS only (no vaccine) or live virus. H&E and immunohistochemical stains for eosinophil major basic protein were performed as described for figure 5. The H&E column is on the left and the Eos MBP column is on the right. Shown are sections from normal mice (no vaccine or live virus) and mice given PBS (no vaccine) or live SARS-CoV and then challenged with SARS-CoV. As shown in the middle and bottom row images, although exposure to SARS-CoV elicits inflammatory infiltrates and accumulation of debris in the bronchial lumen, eosinophils in all groups remain within normal limits.

Representative photo micrographs of lung sections from mice in this experiment two days after challenge with SARS-CoV are shown in figure 5. The pathologic changes were extensive and similar in all challenged groups (H & E stains). Perivascular and peribronchial inflammatory infiltrates were observed in most fields along with desquamation of the bronchial epithelium, collections of edema fluid, sloughed epithelial cells, inflammatory cells and cellular debris in the bronchial lumen. Large macrophages and swollen epithelial cells were seen near lobar and segmental bronchi, small bronchioles and alveolar ducts. Necrotizing vasculitis was prominent in medium and large blood vessels, involving vascular endothelial cells as well as the tunica media, and included lymphocytes, neutrophils, and eosinophils in cellular collections. Occasional multinucleated giant cells were also seen. The eosinophil component of infiltrates was very prominent in animals vaccinated with the experimental vaccine preparations when compared to animals mock-vaccinated using PBS, or those exposed earlier to live virus (figure 6); few to no eosinophils were seen in those lung sections. Thus, while pathology was seen in sections from the control mice, the hypersensitivity-type pathologic reaction with eosinophils was not seen. The morphological identification of eosinophils in H&E stains was supported by using Giemsa stain to highlight intracytoplasmic granules in selected lung sections (not shown), and confirmed by immunostaining with antibodies against mouse eosinophil major basic protein (provided by the Lee Laboratory, Mayo Clinic, Arizona) [36].

The different groups of vaccinated animals showed similar trends in severity of pathology and of eosinophils in inflammatory infiltrates; however, the DIV and BPV preparations at high dosage tended to produce a greater infiltration with eosinophils.

#### Mouse and Vaccine Specificity (Experiment 3)

Experiment 3 was performed to evaluate vaccine and mouse strain specificity. SARS-CoV vaccines used were the DI vaccine (DIV) with and without alum and the bp inactivated vaccine (BPV), which contains alum, at the highest dosage. For mouse strain specificity, Balb/c mice were included for consistency between experiments; C57BL/6 mice were given the same vaccines and dosages as Balb/c mice for comparison as C57BL/6 mice do not exhibit a bias for Th2 immunologic responses as do Balb/c mice [37]–[39]. PBS and live virus controls were again included and trivalent 2010-11 formulation influenza vaccine at a dosage of 12 µg per component was given to assess vaccine specificity.

**Figure 7:**
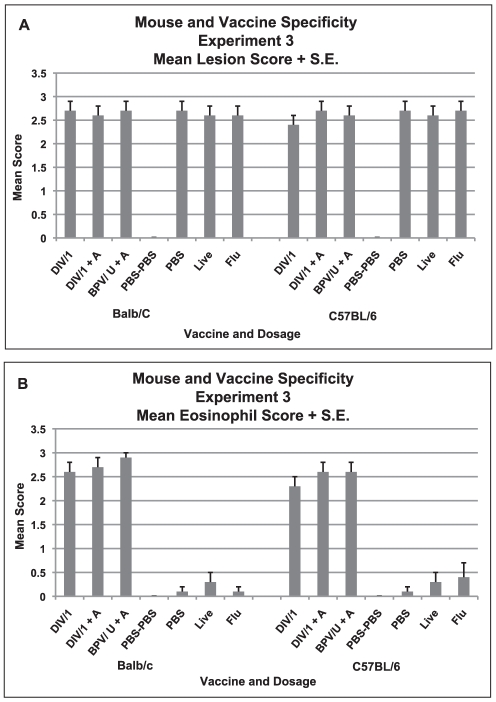
Mouse and Vaccine Specificity, Experiment 3. Serum neutralizing (neut) antibody and lung virus titers for each vaccine dosage group. A. Geometric mean serum antibody titer and standard error of the mean (S.E.) on day 56 for each vaccine dosage group for each mouse strain (Balb/c or C57BL/6). Five mice per group given 0.1 ml of vaccine intramuscularly on days 0 and 28. B. Geometric mean virus titer (log_10_ TCID_50_/g) and standard error of the mean (S.E.) in lungs on day 58 (two days after SARS-CoV challenge for each vaccine dosage group for each mouse strain. Seven to eight mice per group. Vaccines: Double inactivated whole virus, (DIV), β propiolactone inactivated whole virus (BPV), with alum (+A). Analyses: A. GMT for highest DIV dosage without alum greater for Balb/c than C57BL/6 p = .008 but not for alum p>.05. GMT for the BPV vaccine and live virus were not different for the two strains p>.05. B. GMT for PBS control mice were not different p>.05. GMT for DIV without alum and BPV with alum greater for C57BL/6 than Balb/c p = .004.

Neutralizing antibody titers are shown in figure 7A. Geometric mean titers for the highest dose of the DI vaccine were higher for those vaccine groups in the Balb/c mice than the C57BL/6 mice but only the nonadjuvanted DI vaccine group was significantly higher (p = 0.008, Mann Whitney U). The serum antibody responses after BPV and live virus administration were similar for the two mouse strains. After challenge, mean lung virus titers were similar for the PBS control challenged mice of both mouse strains (10^6.7–7.3^ TCID_50_/g lung) (figure 7B). None of the Balb/c mouse groups given either vaccine or live virus earlier yielded virus after challenge but some virus was detected in C57BL/6 mice given the DIV without alum and the BPV with alum (C57BL/6 versus Balb/c, p = 0.004, Mann Whitney U).

**Figure 8:**
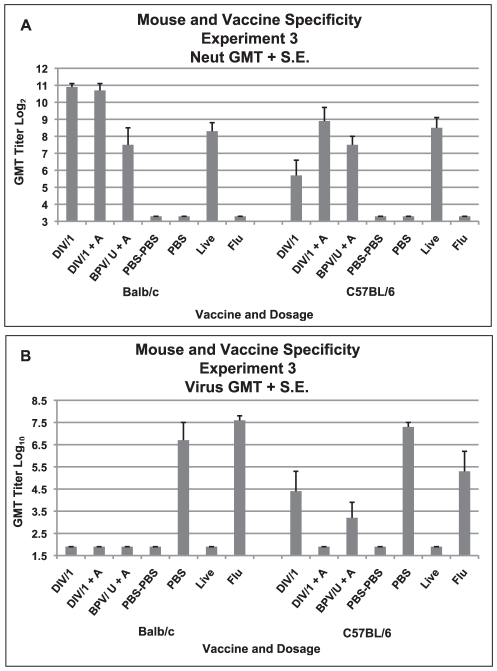
Mouse and Vaccine Specificity, Experiment 3. Mean lung cellular infiltration/lesion pathology and percent eosinophils in infiltrates for each vaccine dosage group for each mouse strain (Balb/c or C57BL/6) two days after challenge with SARS-CoV. A. Mean lesion score and standard error of the mean (S.E.) for each vaccine dosage group. Scores are mean of scores for seven to eight mice per group. Scoring 0 - no definite pathology, 1 - mild peribronchiole and perivascular cellular infiltration, 2 - moderate peribronchiole and perivascular cellular infiltration, 3 - severe peribronchiole and perivascular cellular infiltration with thickening of alveolar walls, alveolar infiltration and bronchiole epithelial cell necrosis and debris in the lumen. Ten to 20 microscopy fields were scored for each mouse lung. B. Mean score and standard error of the mean (S.E.) for eosinophils as percent of infiltrating cells for each vaccine dosage group. Scores are mean of scores for seven to eight mice per group. Scoring: 0 - <5% of cells, 1 - 5–10% of cells, 2 - 10–20% of cells, 3 - >20% of cells. Ten to 20 microscopy fields were scored for each mouse lung. Analyses: A. Mean lesion scores were not different p>.05. B. Mean eosinophil scores were different p<.001. Mean scores for vaccine groups greater than non-vaccine groups for Balb/c and C57BL/6 p<.001 for all comparisons. Mean eosinophil scores for the same groups not different for Balb/c and C57BL/6 p>.05.

**Figure 9:**
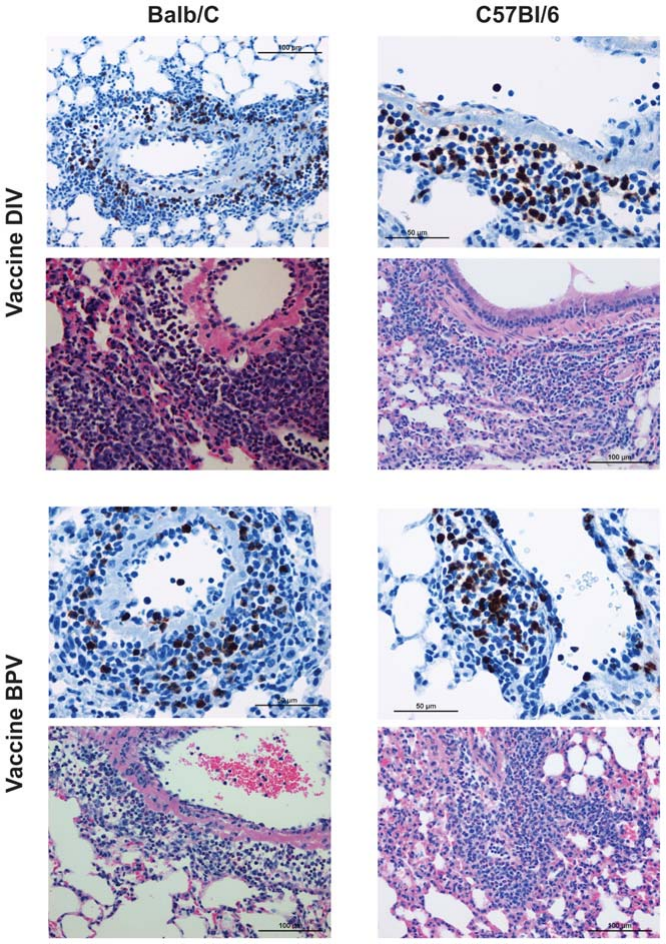
Photomicrographs of Lung Tissue. Representative photomicrographs of lung tissue two days after challenge of Balb/c and C57BL/6 mice that had previously been given a SARS-CoV vaccine. Lung sections were separately stained with H&E (pink and blue micrographs) or the immunohistochemical stain for eosinophil major basic protein (blue and brown micrographs). Balb/c mice lung sections are in the left column and C57BL/6 are in the right column; doubly inactivated whole virus vaccine is in the upper four panels and those from mice given the β propiolactone inactivated whole virus vaccine are in the lower four panels. Pathologic changes observed (inflammatory infiltrates) are similar in Balb/c and C57BL/6 and eosinophils are prominent in both groups.

Mean lung lesion scores two days after challenge were similar for all groups and indicated a moderate to severe degree of cellular infiltration (p>0.05 for each, Anova) (figure 8A). However, eosinophil scores were significantly different between groups (p<0.001, Anova) with significantly lower scores for nonvaccine groups than for vaccine groups of both mouse strains (p<0.001 for all comparable group comparisons, Tukey's HSD). Eosinophil scores for the vaccine groups were not different between the two mouse strains (p>0.05, t test) (figure 8B). Photomicrographs of the different vaccine and mouse strain groups are shown in figure 9. Both vaccines in both mouse strains exhibited significant cellular infiltrations that included numerous eosinophils as shown in the MBP stained sections, a finding consistent with a hypersensitivity component of the pathology. Prior influenza vaccine did not lead to an eosinophil infiltration in the lung lesions after challenge.

## Discussion

The emergence of the disease SARS and the rapid identification of its severity and high risk for death prompted a rapid mobilization for control at the major sites of occurrence and at the international level. Part of this response was for development of vaccines for potential use in control, a potential facilitated by the rapid identification of the causative agent, a new coronavirus [8]–[9]. Applying the principles of infection control brought the epidemic under control but a concern for reemergence naturally or a deliberate release supported continuation of a vaccine development effort so as to have the knowledge and capability necessary for preparing and using an effective vaccine should a need arise. For this purpose, the National Institute of Allergy and Infectious Diseases supported preparation of vaccines for evaluation for potential use in humans. This effort was hampered by the occurrence in the initial preclinical trial of an immunopathogenic-type lung disease among ferrets and Cynomolgus monkeys given a whole virus vaccine adjuvanted with alum and challenged with infectious SARS-CoV [14]. That lung disease exhibited the characteristics of a Th2-type immunopathology with eosinophils in the lung sections suggesting hypersensitivity that was reminiscent of the descriptions of the Th2-type immunopathologic reaction in young children given an inactivated RSV vaccine and subsequently infected with naturally-occurring RSV [32]–[33]. Most of these children experienced severe disease with infection that led to a high frequency of hospitalizations; two children died from the infection [33], [40], [41]. The conclusion from that experience was clear; RSV lung disease was enhanced by the prior vaccination. Subsequent studies in animal models that are thought to mimic the human experience indicate RSV inactivated vaccine induces an increased CD4^+^ T lymphocyte response, primarily of Th2 cells and the occurrence of immune complex depositions in lung tissues [32], [42], [43]. This type of tissue response is associated with an increase in type 2 cytokines including IL4, IL5, and IL13 and an influx of eosinophils into the infected lung; [32], [33], [42], [44]. Histologic sections of tissues exhibiting this type of response have a notable eosinophilic component in the cellular infiltrates. Recent studies indicate that the Th2-type immune response has both innate and adaptive immune response components [33], [43].

In addition to the RSV experience, concern for an inappropriate response among persons vaccinated with a SARS-CoV vaccine emanated from experiences with coronavirus infections and disease in animals that included enhanced disease among infected animals vaccinated earlier with a coronavirus vaccine [31]. Feline infectious peritonitis coronavirus (FIPV) is a well-known example of antibody-mediated enhanced uptake of virus in macrophages that disseminate and increase virus quantities that lead to enhanced disease [31], [45]. Antigen-antibody complex formation with complement activation can also occur in that infection and some other coronavirus infections in animals. Thus, concern for safety of administering SARS-CoV vaccines to humans became an early concern in vaccine development.

As a site proposed for testing vaccines in humans, we requested and were given approval for evaluating different vaccine candidates for safety and effectiveness. Two whole coronavirus vaccines, one rDNA-expressed S protein vaccine and a VLP vaccine prepared by us were evaluated in a Balb/c mouse model, initially described by others, of SARS-CoV [46], [47]. The concern for an occurrence of lung immunopathology on challenge of mice vaccinated with an inactivated virus vaccine, as reported by Haagmans, et al. for ferrets and nonhuman primates, was seen by us after challenge of mice vaccinated with a SARS VLP vaccine [20]. This finding was duplicated in an experiment reported here and was also seen in mice vaccinated with a range of dosages of a double-inactivated whole virus vaccine (DIV) and an rDNA S protein vaccine (SV) although the immunopathologic reaction appeared reduced among animals given the S protein vaccine when compared to those given the whole virus vaccine. In later experiments, these findings were confirmed and the vaccine utilized by Haagmans, et al. was also shown to induce the immunopathology in mice. Thus, all four vaccines evaluated induced the immunopathology; however, all four also induced neutralizing antibody and protection against infection when compared to control challenged animals.

The immunopathology in all experiments in the present study occurred in the absence of detectable virus in lungs of mice two days after challenge with infectious virus. In two experiments, a live virus group subsequently challenged with live virus was included. These challenged animals also exhibited similar histopathologic changes after challenge although no infectious virus was detected in lungs on day two; however, in the latter case, the infiltrates were nearly 100% monocytes and lymphocytes without the eosinophil component seen in the vaccinated challenged animals. In a separate test to assess the effects of the challenge inoculum, mice were given an IN challenge with 10^8^TCID_50_ of inactivated whole SARS-CoV. Lungs of these animals revealed minimal or no histopathologic damage (data not shown). These findings suggest that virus replication probably occurred early after challenge, including in animals given live CoV earlier, and is required for development of pathology, including for the immunopathology. Infection would have been transient, below the limit of detection two days after challenge, or neutralized in lung homogenates before testing for virus.. Nevertheless, the Th2-type immunopathology pattern was seen only in animals given an inactivated vaccine earlier.

During the course of these experiments, a report appeared describing a similar immunopathologic-type reaction with prominent eosinophils in SARS-CoV challenged Balb/c mice that had been given Venezuelan equine encephalitis (VEE) vector containing the SARS nucleocapsid protein gene [18]. Those challenged animals exhibited infection similar to unvaccinated animals as well as Th2-type immunopathology. A similar experiment with a VEE vector containing only the S gene exhibited protection against infection and no immunopathology. More recently, this group has reported immunopathology with prominent eosinophil infiltration after SARS-CoV challenge in Balb/c mice vaccinated with the same double-inactivated whole virus vaccine used in our experiments [28]. They attribute the immunopathologic reaction following these SARS-CoV vaccinations to presence of the nucleocapsid protein (N) in the vaccine.

In another report, vaccinia was used as a vector vaccine for immunizing Balb/c mice with each of the SARS-CoV structural proteins (N, S, membrane, and envelope) and then challenged with SARS-CoV [21]. Virus infection was present in all groups after challenge but reduced in the S vector vaccine group. Histopathology scores were high for the N containing vector group and low for the S containing group and for the vehicle control group. Eosinophilic infiltrates and IL-5 were increased in the N vaccine group but only IL-5 was increased in the S vaccine group.

To be certain the Th2 type immunopathology was elicited by the S protein vaccine in our studies and in hopes a greater immune response would result from higher dosages of the vaccine and induce greater protection against infection as well as reduce or prevent the immunopathology, our experiment 2 used up to 9 µg of the S protein for immunization. While increased titers of serum antibody were induced and no virus was detected day two after challenge in most animals, the Th2-type immunopathology occurred after challenge, and the immunopathology seen earlier after vaccination with the DI whole virus vaccine was seen again. This experiment also included the whole virus vaccine tested earlier in ferrets and nonhuman primates where the Th2-type immunopathology was initially seen. That vaccine, the BPV in this report, exhibited a pattern of antibody response, protection against infection and occurrence of immunopathology after challenge similar to the DI whole virus vaccine (DIV).

A final experiment was conducted to evaluate specificity. The Balb/c mouse was compared to C57BL/6 mice which do not exhibit the Th2 response bias known to occur in Balb/c mice. C57BL/6 mice in that same experiment exhibited results on challenge similar to those seen in Balb/c mice. Challenge of animals given prior influenza vaccine were infected and exhibited histopathologic damage similar to animals given PBS earlier; neither group exhibited the eosinophil infiltrations seen in animals given a SARS-CoV vaccine.

In these various experiments alum was used as an adjuvant and this adjuvant is known to promote a Th2 type bias to immune responses [48]. However, the immunopathology seen in vaccinated-challenged animals also occurred in animals given vaccine without alum. In an effort to determine whether an adjuvant that induced a bias for a Th1-type response would protect and prevent the immunopathology, we initiated an experiment where the DI PBS suspended vaccine was adjuvanted with Freund's complete adjuvant, a Th1-type adjuvant. However, this experiment was aborted by the September, 2008, Hurricane Ike induced flood of Galveston, Texas. An experiment with a SARS-CoV whole virus vaccine with and without GlaxoSmithKline (GSK) adjuvant ASO1 in hamsters has been reported [25]. This adjuvant is thought to induce Th1-type immune responses [49]. The authors indicate no lung immunopathology was seen among animals after challenge, including the group given vaccine without adjuvant; however, whether the hamster model could develop a Th2-type immunopathology is uncertain. Finally, a number of other studies of vaccines in animal model systems have been reported but presence or absence of immunopathology after challenge was not reported.

A summary of the SARS-CoV vaccine evaluations in animal models (including the current report) that indicated an evaluation for immunopathology after challenge is presented in Table 2. As noted all vaccines containing S protein induced protection against infection while the studies with VEE and vaccinia vector containing the N protein gene only did not. Also shown is that a Th2-type immunopathology was seen after challenge of all vaccinated animals when evaluation for immunopathology was reported except the study in hamsters with a GSK whole virus vaccine. Thus, inactivated whole virus vaccines whether inactivated with formalin or beta propiolactone and whether given with our without alum adjuvant exhibited a Th2-type immunopathologic in lungs after challenge. As indicated, two reports attributed the immunopathology to presence of the N protein in the vaccine; however, we found the same immunopathologic reaction in animals given S protein vaccine only, although it appeared to be of lesser intensity. Thus, a Th2-type immunopathologic reaction on challenge of vaccinated animals has occurred in three of four animal models (not in hamsters) including two different inbred mouse strains with four different types of SARS-CoV vaccines with and without alum adjuvant. An inactivated vaccine preparation that does not induce this result in mice, ferrets and nonhuman primates has not been reported.

**Table 2:** Summary of Reported Protection and Immunopathology in Animal Model Studies with SARS Coronavirus Vaccines.

Animal Model	Vaccine^1^	Protection^2^	Immunopathology^3^
Mice	Whole virus^tr^		
	w alum	Yes	Yes
	Whole virus^25,tr^		
	w alum	Yes	Yes
	wo alum	Yes	Yes
	VLP^17,tr^		
	w alum	Yes	Yes
	wo alum	Yes	Yes
	S Protein^tr^		
	w alum	Yes	Yes
	wo alum	Yes	Yes
	VEE Vector^15^		
	for N protein	No	Yes
	for S protein	Yes	No
	Vaccinia vector^18^		
	for N protein	No	Yes
	for S protein	Yes	?No
Ferrets	Whole virus^11^		
	w alum	Yes	Yes
Nonhuman Primate^4^	Whole virus^11^		
	w alum	Yes	Yes
Hamsters	Whole virus^22^		
	w ASO1	Yes	No

1 Reference for each indicated; tr = this report; w = with, wo = without.

2 Protection against infection (reduced lung virus after challenge).

3 Th2-type immunopathology as indicated by cellular infiltrates with prominence of eosinophils.

4 Cynomolgus monkeys.

This combined experience provides concern for trials with SARS-CoV vaccines in humans. Clinical trials with SARS coronavirus vaccines have been conducted and reported to induce antibody responses and to be “safe” [29], [30]. However, the evidence for safety is for a short period of observation. The concern arising from the present report is for an immunopathologic reaction occurring among vaccinated individuals on exposure to infectious SARS-CoV, the basis for developing a vaccine for SARS. Additional safety concerns relate to effectiveness and safety against antigenic variants of SARS-CoV and for safety of vaccinated persons exposed to other coronaviruses, particularly those of the type 2 group. Our study with a VLP SARS vaccine contained the N protein of mouse hepatitis virus and Bolles, et al., reported the immunopathology in mice occurs for heterologous Gp2b CoV vaccines after challenge [25]. This concern emanates from the proposal that the N protein may be the dominant antigen provoking the immunopathologic reaction.

Because of well documented severity of the respiratory disease among infants given an inactivated RSV vaccine and subsequently infected with RSV that is considered to be attributable to a Th2-type immunopathologic reaction and a large number of studies in the Balb/c mouse model that have described and elucidated many components of the immunopathologic reaction to RSV vaccines, the similarity to the SARS-CoV vaccine evaluations in Balb/c mice supports caution for clinical vaccine trials with SARS-CoV vaccines in humans. Of interest are the similar occurrences in C57BL/6 mice and in ferrets and nonhuman primates that provide alternative models for elucidating vaccine-induced mechanisms for occurrences of Th2 immunopathologic reactions after infection. As indicated, strong animal model evidence indicates expression of the N protein by SARS-CoV vector vaccines can induce sensitization leading to a Th2–type immunopathology with infection. In contrast to our results, those studies did not find clear evidence of the Th2 type immunopathology on challenge of mice given a vector vaccine for the S protein. The finding of a Th-2-type pathology in our studies in animals immunized with an rDNA-produced S protein is unequivocal. In this regard, animal model studies with FIPV in cats and RSV in mice have indicated that viral surface proteins may be the sensitizing protein of inactivated vaccines for immunopathology with infection [32], [45]. This suggests that presentation of the S protein in a vector format may direct immune responses in a different way so that sensitization does not occur.

Limitations of the present studies include their performance in mice only and uncertainty of the relevance of rodent models to SARS-CoV vaccines in humans. Additionally, a more intense study for virus replication including quantitative RT-PCR assays might have confirmed the probability that virus replication is required for induction of the immunopathology after vaccination. Evaluations of mechanisms for the immunopathology, including immunoglobulin and cytokine responses to vaccines and tests for antigen-antibody complexes in tissues exhibiting the reaction, could have strengthened the Th2-type immunopathology finding. Finally, a successful study with a Th1-type adjuvant that did not exhibit the Th2 pathology after challenge would have confirmed a Th2 bias to immune responses as well as provide a potential safe vaccination approach for SARS.
