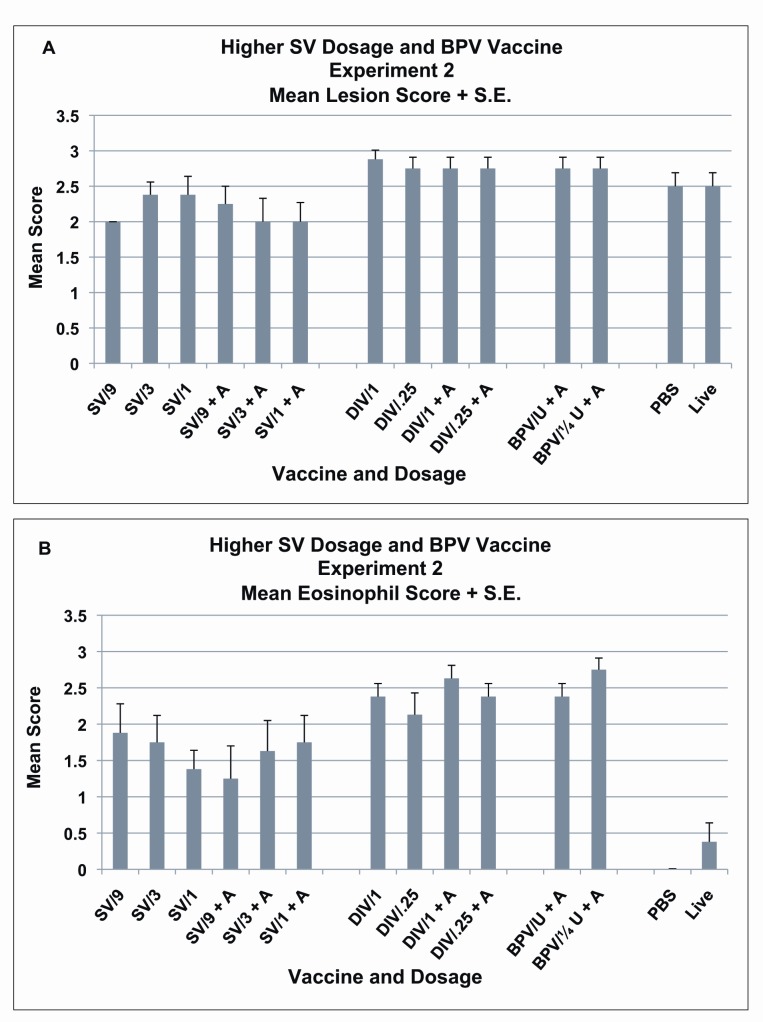# Correction: Immunization with SARS Coronavirus Vaccines Leads to Pulmonary Immunopathology on Challenge with the SARS Virus

**DOI:** 10.1371/annotation/2965cfae-b77d-4014-8b7b-236e01a35492

**Published:** 2012-08-09

**Authors:** Chien-Te Tseng, Elena Sbrana, Naoko Iwata-Yoshikawa, Patrick C. Newman, Tania Garron, Robert L. Atmar, Clarence J. Peters, Robert B. Couch

There were errors in Figures 1, 2, and 4. The correct figures can be viewed here:

Figure 1 

**Figure:**
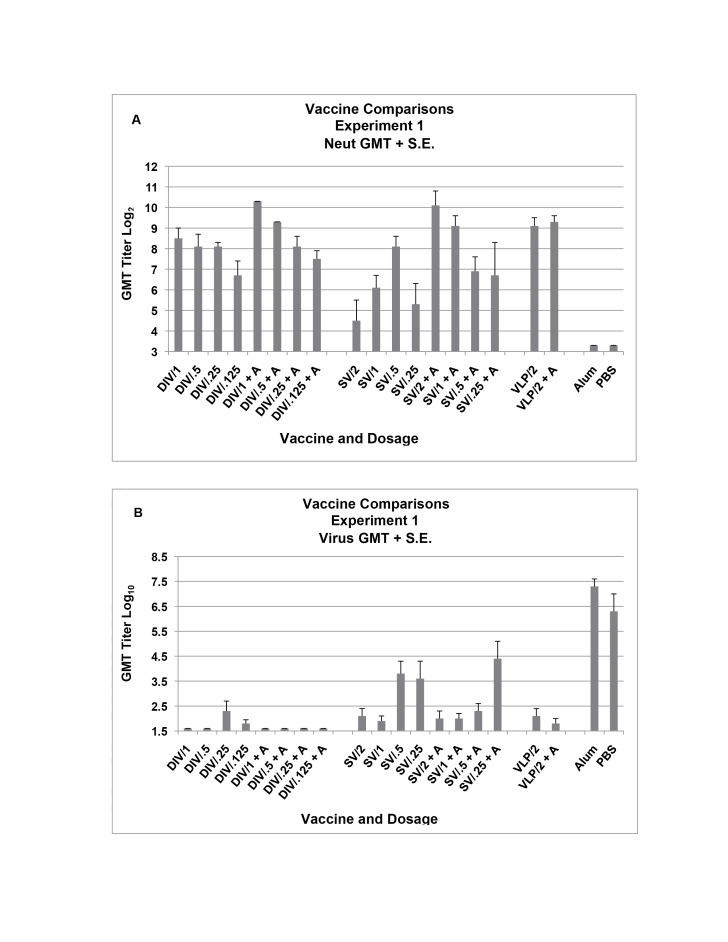


Figure 2 

**Figure:**
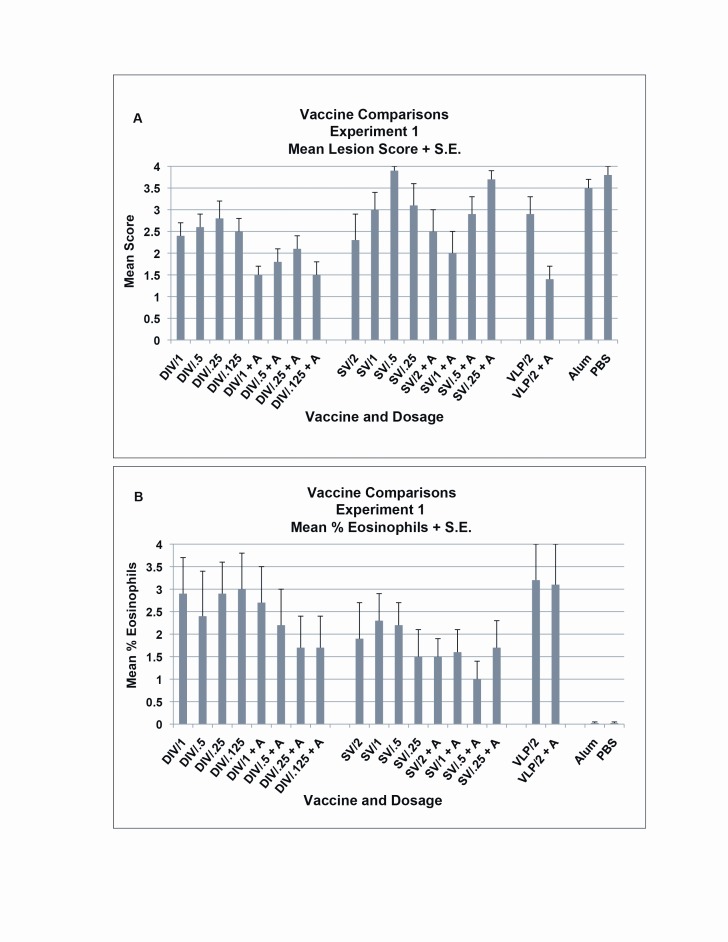


Figure 4 

**Figure:**